# First person – Jocelyn Wessels

**DOI:** 10.1242/dmm.042481

**Published:** 2019-10-01

## Abstract

First Person is a series of interviews with the first authors of a selection of papers published in Disease Models & Mechanisms (DMM), helping early-career researchers promote themselves alongside their papers. Jocelyn Wessels is first author on ‘Medroxyprogesterone acetate alters the vaginal microbiota and microenvironment in women and increases susceptibility to HIV-1 in humanized mice’, published in DMM. Jocelyn conducted the research described in this article while a Postdoctoral Fellow in Dr Charu Kaushic's lab at McMaster University, Hamilton, Canada. She is now a Postdoctoral Fellow in the lab of Dr Nicholas Leyland at McMaster University, investigating women's reproductive health, in particular reproductive microbiotas.


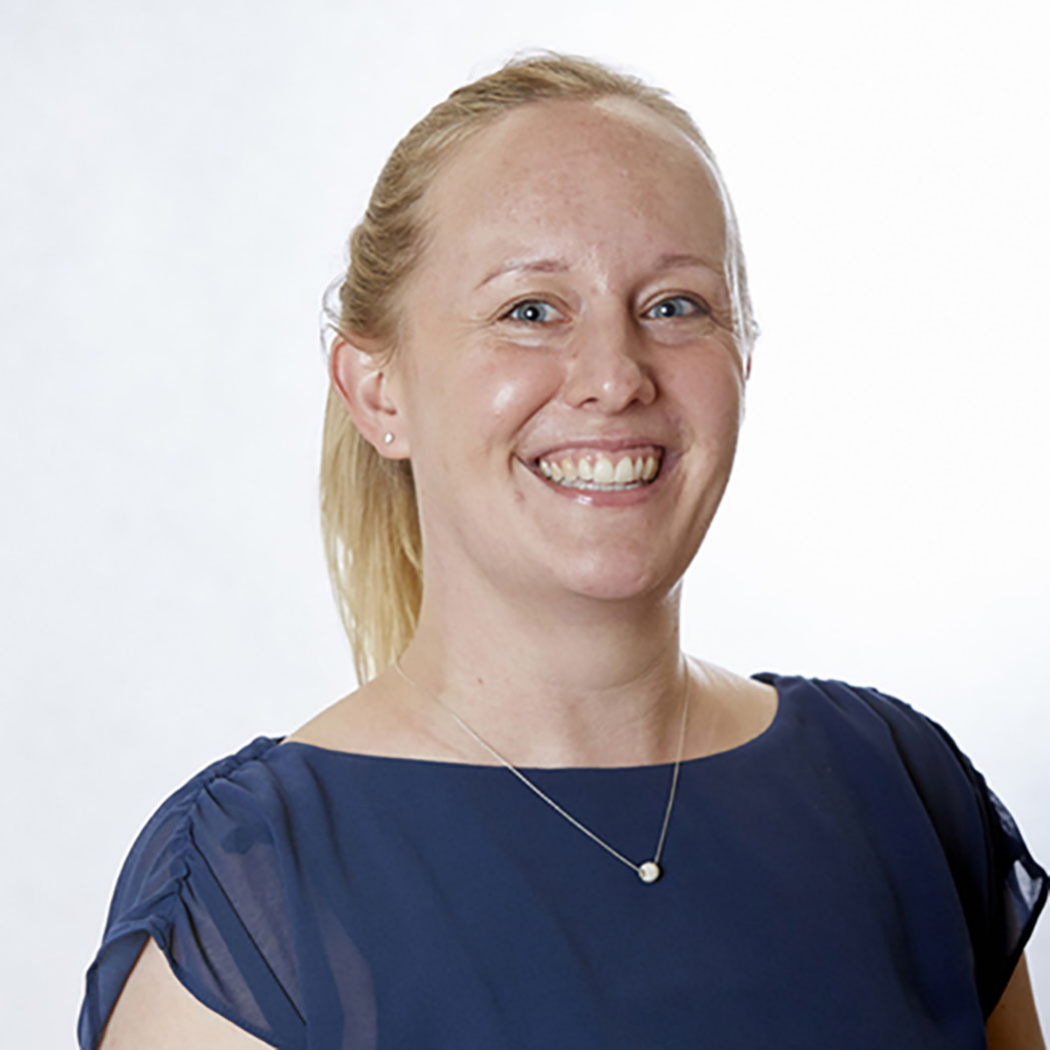


**Jocelyn Wessels**

**How would you explain the main findings of your paper to non-scientific family and friends?**

The hormonal contraceptive medroxyprogesterone acetate (MPA) appears to increase the risk of women contracting human immunodeficiency virus (HIV). Similarly, the bacteria residing in the vagina can modify a woman's risk. In this paper, we asked whether MPA changed the bacteria in the vagina, whether it changed the sugars present in the vagina and whether mice with a human immune system were more likely to contract HIV if they were given MPA. We found that MPA increased the diversity of the bacteria present in the vagina and that it lowered a sugar called glycogen (in humans and in mice), suggesting that MPA does change the bacteria and environment of the vagina. We also showed that mice with a human immune system that were given MPA had a higher rate of HIV infection when we exposed them to the virus, compared with mice that were not given MPA. This suggests that one way MPA may impact the risk of HIV in women is by changing the vaginal environment.

**What are the potential implications of these results for your field of research?**

The potential implications of the ability of MPA to alter the bacteria in the vagina and the sugars in the vaginal microenvironment are that this may provide another mechanism by which MPA may be able to modify a woman's risk of acquiring HIV. The bacteria in the vagina use carbohydrates such as glycogen as a source of energy and changing the substrates available to them likely changes which types of bacteria can survive under the new conditions. Other studies have found certain community state types of bacteria to be associated with increased susceptibility to HIV, suggesting that the bacteria present in the vagina can affect the risk of contracting HIV. In addition, this manuscript is the first report of an increased rate of intravaginal HIV infection in humanized mice treated with MPA.

“This manuscript is the first report of an increased rate of intravaginal HIV infection in humanized mice treated with MPA”

**What are the main advantages and drawbacks of the model system you have used as it relates to the disease you are investigating?**

Humanized mice are a good tool to study HIV infection as they have human HIV target cells (T cells, dendritic cells and macrophages) in their peripheral circulation and tissues. However, as with any model system, there are drawbacks. For example, the humanized mice are immunodeficient and we are not sure how functional the engrafted human immune cells are. Therefore, it is unclear whether viral infection and dissemination proceed in the humanized mice in the same manner as they would in humans.

**What has surprised you the most while conducting your research?**

I was surprised that vaginal glycogen is lower in both women and humanized mice that had been given MPA. Perhaps it should not have come as such a surprise but, at the time, my hypothesis had been that there would not be a difference. It is always surprising when hypotheses are incorrect, at least to me!

**Figure:**
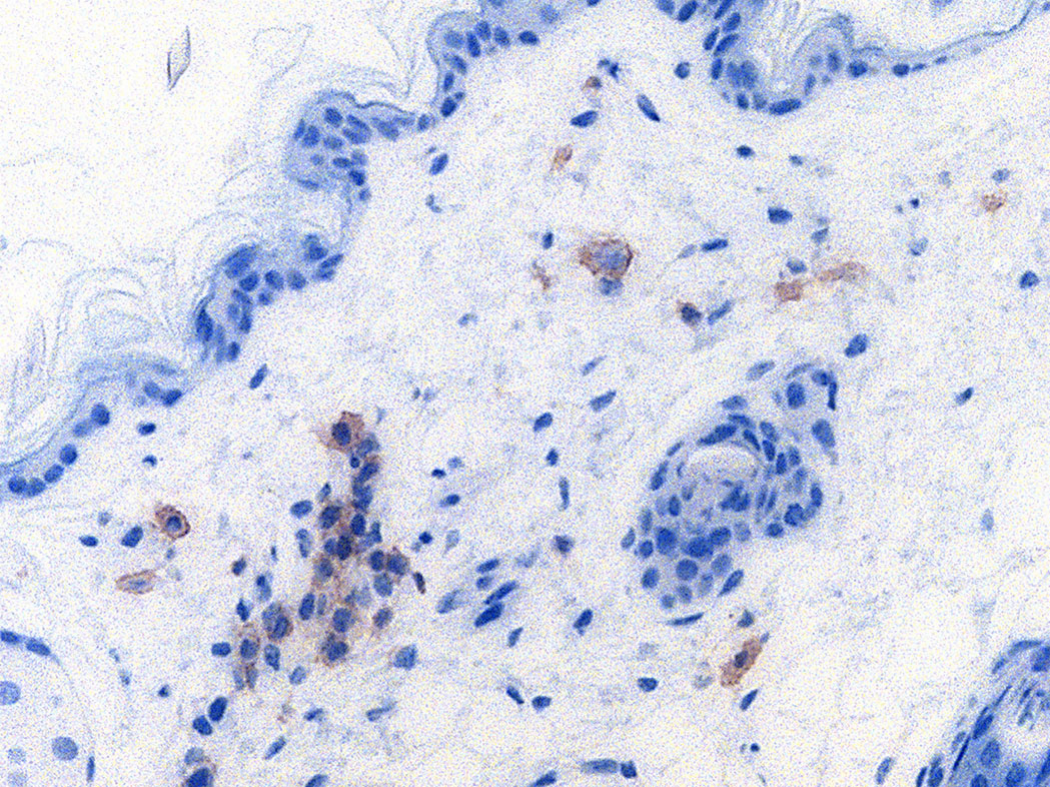
**Human CD4+ cells (HIV target cells, brown) in the vaginal stroma of a humanized mouse.**

**Describe what you think is the most significant challenge impacting your research at this time and how will this be addressed over the next 10 years?**

I think the most significant challenge impacting this field of research is that just because we can describe the bacteria that are present in the vagina, it does not mean we understand their function(s) or how they interact with each other. I think some of this will be addressed in the next 10 years using bacterial co-culture with cells, and other functional assays/data mining to estimate the functions of bacterial communities.

“It is always surprising when hypotheses are incorrect, at least to me!”

**What changes do you think could improve the professional lives of early-career scientists?**

I have been very fortunate to have good mentors; something that I think if it were more formally available might really improve the professional lives of early-career scientists. Also, additional funding to bridge the gap between being a Postdoctoral Fellow and Faculty would help!

**What's next for you?**

I am presently working on a second Postdoctoral Fellowship at McMaster University and am interested in the endometrial microbiota and endometriosis. Stay tuned…
